# Quantification of biological variation in blood‐based therapy – a summary of a meta‐analysis to inform manufacturing in the clinic

**DOI:** 10.1111/vox.12288

**Published:** 2015-07-14

**Authors:** J. A. Thurman‐Newell, J. N. Petzing, D. J. Williams

**Affiliations:** ^1^ Healthcare Engineering GroupCentre for Biological EngineeringLoughborough UniversityLoughboroughUK

**Keywords:** biological variation, blood, HSCT, process design, quality control

## Abstract

**Background and Objectives:**

Biological raw materials, the basis for cellular therapies such as stem cells, have a significantly greater degree of complexity than their traditional pharmaceutical counterparts. This can be attributed to the inherent variation of its source – human beings. Currently, cell therapies are made in small, *ad hoc* batches, but larger scale production is a prerequisite to meeting future demand and will require a quality‐by‐design approach to manufacturing that will be designed around, or be robust to this variation. Quantification of variation will require understanding of the current baseline and stratification of its sources.

**Materials and Methods:**

Haematopoietic stem cell therapy was chosen as a case study to explore this variation, and a PRISMA‐guided (Preferred Reporting Items for Systematic Reviews and Meta‐Analyses) systematic meta‐analysis was carried out for a number of predetermined cell measurements.

**Results:**

From this data set, it appears that the extent of variation in therapeutic dose (in terms of transplanted total nucleated cells and CD34^+^ cells per kilogram) for HSCT is between one and four orders of magnitude of the median.

**Conclusions:**

This is tolerated under the practice of medicine but would be unmanageable from a biomanufacturing perspective and raises concerns about comparable levels of efficacy and treatment. A number of sources that will contribute towards this variation are also reported, as is the direction of travel for 4 greater clarity of the scale of this challenge.

## Context

Biological raw materials, the basis for cellular therapies (CTs) such as stem cells, have a significantly greater degree of complexity, sensitivity and plasticity than their more traditional pharmaceutical counterparts. This can be attributed to the inherent variation of its source – human beings.

Currently, stem cell therapies are wrought in small batches within a hospital/laboratory environment on an *ad hoc* basis, regulated as either for homologous use or minimally manipulated 1. Larger scale production is a prerequisite to meeting future clinical demand and will require a quality‐by‐design manufacturing process that is either designed around the inherent biological variation of the raw material (and its sensitivity during the process) or be robust to the variation.

A product (the cell in this case) is traditionally manufactured to a specification provided by the prescriber. This would include a number of tolerances – the amount of change the product can undergo and still remain functional to the predetermined specification. The current tolerances for cellular therapies, such as blood‐based haematopoietic stem cells, are based on a minimum and optimal threshold criteria; for example, in autologous HSCT derived from peripheral blood, the minimum is considered to be 2 × 10^6^ cells/kg bodyweight and the optimum considered to be 5 × 10^6^ cells/kg bodyweight 2, 3. Reducing variation decreases the number of defects within the product line, and increases its overall quality at a reduced cost – for cellular therapy, this would mean maximizing patient longevity and quality of life while minimizing costs. For CT, this means a consistent, quality product with a known efficacy at scale. Variation is never eliminated entirely, and consists of two broad categories; common cause and special cause variation. Common cause variation is expected inherent variation as a function of the raw material and the process involved. Special cause variation is unexpected variation due to external factors or unaccounted variables – such as machine failure causing a product to deviate from tolerance. A process with only common cause variation is stable and predictable.

Quantifying variation will require elucidation of the baseline variation for the process input/output, and identification of causes of variation within this process, and their magnitude. This will identify critical‐to‐quality attributes and measurands that are key contributing factors towards the quality and efficacy of the final product and determine the extent of common and special cause variation. As Lord Kelvin stated 100 years ago and still holds true today;

…when you can measure what you are speaking about, and express it in numbers, you know something about it; but when you cannot express it in numbers, your knowledge is of a meagre and unsatisfactory kind 4 .

As pharmaceuticals are defined in terms of grams per kilogram and purity of active ingredient, so must HSCTs. Blood‐based therapies are characterized by their cell content, specifically cells per kilogram of patient weight, and may include total nucleated cells (TNC) and CD34^+^ cells. The number of TNCs/product is the more traditional, commonly reported parameter for cell dose and represents the number of cells present excluding red blood cells and platelets. A more specific characterization uses the cluster of designation (CD) cell surface marker system. CDs are involved in critical cellular functions and are therefore indicative of particular cell types, enabling the identification of specific cell types. CD34 is a particular marker found on haematopoetic progenitor cells (although not all CD34^+^ cells are HPCs 5).

Due to the prevalence of HSCT, it is an ideal exemplar to benchmark the variation prevalent within a cellular therapy using a transplant. Additionally, as HSCT is primarily minimally manipulated, it has the potential to act as a case study to inform the design of processes for more complex, future biological manufacturing with higher regulatory thresholds than transplants and also importantly these materials may also form the starting material for a therapeutic with a higher regulatory threshold.

## Establishing a baseline

Considering the anecdotal evidence encountered by this team of the extent of biological variation encountered, a number of exploratory investigations were devised to gather the experiences of clinical, industrial and academic bodies, evaluate the extent of the challenge and inform the methodology for the next, data‐driven steps.

**Figure 1:**
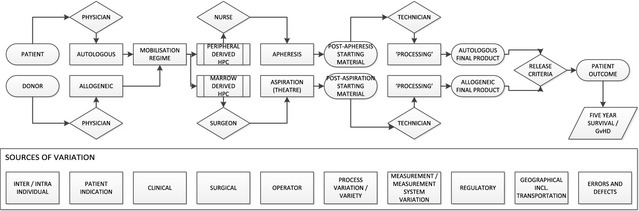
A generic process map for haematopoetic stem cell therapy.

One outcome of these investigations has been the production of a generic process map for HSCT (Fig. 1) that illustrates the universal typical procedures currently followed in the hospital/laboratory environment. This diagram illustrates a number of potential sources of variation, but a greater degree of resolution as to the extent and distribution of variation within this process is required. As a result a number of clinical and open‐source data sets were identified for data mining – one of which was ascertained as the medical literature available in the public domain.

## Systematic literature meta‐analysis

Although not specifically designed to be utilized in this manner, or for this particular issue, it was hypothesized that sufficient information may be present within the medical literature to complete a preliminary investigation into the extent of biological variation – sources, collected cell characteristics, product/dose characteristics, process specifics and patient outcomes – if the nature of such a data set and its caveats were understood. The results of this investigation would identify key areas of interest, aspects requiring further refinement, and promote critical process development discussion between industrial, clinical and academic bodies.

This systematic meta‐analysis aims to examine the literature surrounding HSCT for a number of key predetermined variables. The primary objective of this analysis has been to gain a baseline understanding as to the extent of biological variation in collected and transplant cell metrics. Secondary objectives were data‐dependant and included the effect of different processes, indications and donor/patient characteristics on the aforementioned cell metrics.

## Methodology

This meta‐analysis was guided by the principles prescribed by the preferred reporting items for systematic reviews and meta‐analyses statement (PRISMA 6).

Web of Science, an online database and education resource, was used to search the literature, using a number of predetermined keywords, medical search headings (MeSH) and publication dates (see Table 1). Articles were restricted to English language unless a native translation was provided, and only refereed journals were used (conference proceedings were excluded, for example). The abstracts of the resultant studies were then screened for likelihood of containing cell data – for example those that were comparison/outcome studies or clinical trials. Eligible publications were obtained in full and examined manually for patient, donor and graft characteristics guided by Table 2. These characteristics were identified by previous discussion and mind‐mapping. The primary characteristics were mandatory for studies to pass through to the data extraction stage. Qualitative data such as methodology (where recorded) were reduced to single word/numerical data. The name of the first author and a unique identification number were used to mark papers used for future reference.

**Table 1:** Methodology of the Meta‐Analysis search

	Search Terms	Search Results	Of which contained data
A	Autologous haematopoetic stem cell therapy (2003–2013)	298	31
B	Autologous haematopoetic stem cell therapy (1992–2002)	105	6
C	Allogeneic haematopoetic stem cell therapy (2003–2013)	1183	36
D	Allogeneic haematopoetic stem cell therapy (1992–2002)	125	4
E	Stem cell comparison (2003–2013)	585	9
F	Stem cell outcome (2003–2013)	894	40
		3190	126

**Table 2:** Table containing the list of predetermined variables that each study was screened for

Primary Study Characteristics	Secondary Study Characteristics	Tertiary Study Characteristics
Number of Donors	Donor Mobilisation Drug	Named Collection Equipment
Donor Gender	Donor Mobilisation Regime	Named Processing Equipment
Donor Age^a^	Day of Aspiration/Apheresis	Named Analytical Equipment
Donor Weight (kg)^a^	Study Start and End Year	CD34 Elucidation Method
Donor Ethnicity	Number of Centres involved in Study	Number of Aspirations/Apheresis procedures per donor
Number of Patients	Country of Study	Apheresis flow rate used (ml/min)
Patient Gender	Patient Indication	Target Apheresis Volume (ml)
Patient Age^a^	Patient Prior Medication	Duration of Apheresis
Patient Weight (kg)^a^	Patient Prior Stem Cell Therapy (Yes/No)	Target Apheresis CD34^+^ Count
Patient Ethnicity		Number of times Donor Complete Blood volume was processed
Patient Conditioning		Number of Grafts/Transfusions per patient
Autologous or Allogeneic Therapy		Collection aims for TNC, MNC and CD34^+^ cell populations
Source of Stem Cells (marrow, peripheral, cord or mixed)		Volume of Collection (ml/kg)
COLLECTED TNC, MNC, CD34^+^, CFU‐GM and viability (mean, median, standard deviation, upper and lower ranges)		
TRANSPLANTED TNC, MNC, CD34^+^, CFU‐GM and viability (mean, median, standard deviation, upper and lower ranges)		

a At time of procedure.

As a single operator was used to carry out this methodology, there may exist a bias, which may have excluded data‐containing studies. A number of these will also be missing due to insufficient search terms, limitations of the database used and data published in other languages.

Data were extracted from the full article into a spreadsheet within Microsoft Excel. Both Excel and IBM spss 22.0 (New York) were used for data analysis. Literature that yielded data was downloaded and stored for future record alongside its unique identification number.

## Results and discussion

The primary output of this meta‐analysis was a number of diagrams that demonstrate the extent of variation in cell dose found within the literature. There were insufficient data within the meta‐analysis sources to produce a similar demonstration for cell content of the raw materials. TNC and CD34^+^ cell count were the most prevalent cell characteristic reported.

**Figure 2:**
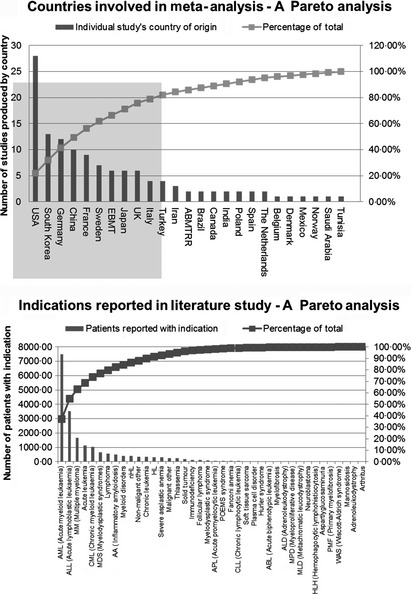
Pareto analyses of meta‐analysis database by geographical location and patient indication.

Data drawn from this meta‐analysis originated from multiple global sources, clinical centres, clinicians/surgical teams, addressing different indications and derived from different patient and donor demographics. Stratification into each of these subsets was not possible due to the limitations of the literature; however, the data set was scrutinized using Pareto analysis as a factor of location and indication of study. Pareto analyses are one of the seven tools of quality in the manufacturing sector, the Pareto rule being commonly referred to as the 80/20 rule – the observation that 20% of the causes determines 80% of the problems. In this case, each variable (indication/country) is plotted in descending order from highest to lowest contribution with an overlay of percentage cumulative contributions (Fig. 2).

Figure 2 illustrates strong contributions to this data set from US, German, Chinese and South Korean contributors. Additionally, the majority of HSCT applications focused on leukaemia as a target indication. This indication has led to the identification of a number of substantial clinical data sets that could provide statistically significant conclusions at a higher resolution.

**Figure 3:**
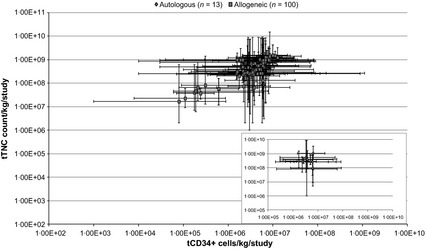
Variation in therapeutic dose: The median TNC count against median CD34^+^ cell count transplanted in each study.

Figure 3 plots the median TNC count against the median CD34^+^ cell count of the given cell dose for a given literature study. The range in dose given to patients within each study is represented by the lines spreading out from these points and has been drawn on a logarithmic scale. Fig. 3 is limited to those studies that provided both median and range data for both TNC count and CD34^+^ cell count. This has been further stratified into autologous and allogeneic therapy, as annotated by the legend.

This key graph emphasizes that the variation in HSCT dose in any given study from this data set can be between one and four orders of magnitude around the median. To illustrate this using a more traditional example, one and four orders of magnitude are the equivalent of giving a dose of between 1 × 10^6^ cells/kg and 1 × 10^10^ cells/kg.

This difference in cell dose raises concerns about comparable levels of efficacy and treatment within individual studies and will only complicate attempts to discern the definitive mode of action for HSCT and the dose/response relationship. When it comes to larger scale production, this level of variation would not permit a stable and predictable manufacturing process and would be unacceptable within an equivalent non‐biological process.

However, there are a number of considerations regarding this data set that must be taken into account

- Limited data reported in the given studies meant that stratification according to patient indication or demographic, and donor metrics such as age, weight or ethnicity were not possible. To identify any common and special cause variation due to donor/patient metrics, further stratification that would allow this was not possible.
- Cell dose was inconsistently reported as either cells/kg patient or donor bodyweight. Due to the presence of paediatric donors/patients, a number of datapoints may be skewed.
- Methodology was rarely reported, including isolation technique, patient mobilization drug/regime/conditioning and apheresis procedure for example. Considering the significant effect, these can have on the cell content of the raw input material; these are important variables to stratify the effect of differing processes on the variation.
- Whether fresh or cryopreserved cells were used was unclear in a number of cases; a further important stratification in the context of product efficacy and yield postcryopreservation.

However, the substantial variation encountered in Fig. 3 can be attributed to a number of broad categories (albeit at an unknown magnitude and detail).

## Sources of variation

- Intra‐individual or ‘Within‐Person’ Variation – This is the effect of changes within a specific donor due to certain internal or external circumstances, such as changes in blood composition due to seasonality, circadian rhythm, exercise, extent of specific illnesses and the particular method of isolating the raw material 7, 8.
- Inter‐individual or – ‘Between‐Person – This is the most visually apparent of sources and concerns the differences between individuals such as age, weight, lifestyle choice and ethnicity 9, 10, 11. The scarcity of donor interindividual variables meant that these were grouped together, which may add to the overall variation found in Fig. 3.
- Clinical Variation – The previous two sources are variation expected during both the extraction of HPCs and their application as a treatment. However, this assumes that the best practice is being applied, and where this is not the case – such as when protocols or techniques are out of date, or if limitations in resources are apparent – then the difference between practice used and the best practice available worldwide is known as ‘unwanted variation’^12^, ^13^. This is highly dependent on the skills and resources of the particular therapeutic centre and is sensitive to geographical variation (see below). This type of variation is particularly important from a patient perspective and the pursuit of clinical trials, as this leads to variation in the quality of treatment and the potential outcome and survival chances of the patient 14.
- In Theatre Variation – This instance specifically applies to bone marrow aspiration, as this requires an invasive procedure. This source has been separately defined to clinical variation as it is specifically focused on the techniques, tools and methods used by individual surgeons to isolate and transplant cellular material and has a similar effect on clinical outcome 15. For HSCT, this is specifically the isolation techniques of the raw material and the application of the final therapeutic via transfusion. Isolation of raw material is a particularly important step, as higher the quality of the raw material, the higher the quality of the product.
- Transplant Type – Depending on the source of the cells, HSCT is either referred to as autologous or allogeneic. Autologous HSCT uses starting materials from the patient and is currently the least expensive 16. Allogeneic HSCT uses starting materials from another matched related or unrelated donor and by not being patient specific has potential for large‐scale manufacture 17. Allogeneic sources will tend to be in a healthier state than an equivalent autologous source, so this may have implications for cell quality. However, the allogeneic source must be tissue compatible with the patient.
- Stem Cell Source – The three current main sources of HSCs are peripheral and cord blood, and bone marrow. Peripheral blood is the most common source in Europe (99% autologous, 71% allogeneic), with bone marrow second (1% autologous, 22% allogeneic) and cord blood third (6% allogeneic)^18^. Each has their own distinct advantages and foibles. Bone marrow has a higher risk to the donor that increases with age, requiring multiple aspirations from multiple sites to obtain optimal numbers 19 and can be painful. Peripheral blood is comparably more convenient for the donor as apheresis avoids anaesthesia and theatre. Peripheral blood products tend to have more CD34^+^ cells present than comparable bone marrow 20, but this is derived from a longer procedure than the single extraction session typical with bone marrow sourced material. Additionally, peripheral blood has a higher incidence of acute and chronic graft‐versus‐host disease 21. Both marrow and peripheral blood have longer transplant waiting times than cord blood but allow for multiple donations, whereas cord blood is a single donation. Cord blood has the lowest volume and cell number (although CD34^+^ cells from cord blood have been reported to be more proliferative 22) but has a low donor risk and GvHD incidence.
- Operator Variation – Cell culture has been described as not unlike cooking or gardening 23 and is reminiscent of when products were manufactured by arts transferred by the historical Master and Apprentice system. HPC product processing is not dissimilar to this and can be further split into three subcategories.
  - *Inter‐operator variance* – This is the differences between individual operators when applying the same methodology (usually following a standard operating procedure). SOPs define a specific way of working, and this variation is the difference in the product due to how these SOPs are interpreted and carried out between different operators. This can be a factor of training and inate understanding of the process by the individual.
  - *Intra‐operator variance* – This occurs when the mood or motivation of a particular operator affects the product 24. This may be due to a specific time of day, or life event of the individual that has a negative or positive affect on their quality of work or adherence to SOPs.
  - *The Effect of Learning* – This is improvement over time due to increase in the competency/skill of the operator 25. As HSCT products are produced by particularly manual processes, this is a particular important source, as many key process steps are directly controlled by a human operator; during separation of blood using a cell processor for example.
- Human versus Machine Variation – One of the next steps in controlling operator variance is the use of automation to reduce the human element and improve product quality. Machine operators are not affected by inter‐ or intra‐operator variance. There are a number of technological hurdles to climb before fully automated cellular product manufacturing, but robotic operators have been shown to reduce the effects of variation present in biological processes, compared to manual human operators 26.
- Geographical Variation – Due to varying sources of funding, knowledge and experience of different medical schools, and access to equipment/facilities, there can be variation in the process method and product application of the ‘same’ therapy between different clinical centres within a country, or between countries 27. Clinical, surgical and operator variance are all factors of geographical variation, of which will be a key factor during and after the roll‐out of a therapeutic to more than one country. Another facet of geographical variation is the challenges surround product shelf life and transport of product from ‘factory’ to clinical location and the effect this will have on the its efficacy as a result, with cryopreservation being a particular issue in this instance. In its current iteration, HSCT either deals with either fresh product isolated and transfused within 72 h, chilled between 1°C and 10°C for transportation or cryopreserved and transported or stored.
- Regulatory Variation – The difference in requirements for a commercial therapeutic (and therefore the process and standards required to manufacture) can vary between regulatory bodies.
- Process Variation/Variety of Processes – The variation inherent to the process, due to the protocol and/or equipment used, or the variation between differing processes and could be due to the use of differing machines, settings on the same machine, how the process is designed and how suboptimal the process is. In engineering, this is assessed by applying an engineering tolerance to each product attribute (‘tolerance stacking’), which is the variation a particular measurement cannot exceed otherwise the product is out of specification. In *Rivadeneyra‐Espinoza et al*.^28^ *,* the enumeration of CD34^+^ cells within the same laboratory, but using different instruments or protocols produced significantly different numbers of cells.
- Measurement/Measurement system Variation – This has two key components, precision and accuracy of the measuring system/equipment. Accuracy represents how close the measured value is to the ‘true value’. The true value will often be expressed in the form of a physical attribute that is time invariant under controlled conditions. This may be a challenge for a biological process. Precision represents the degree to which measurements can be repeated, under identical conditions, and produce the same value. Measurement resolution is an additional component that must be taken into account with the increased complexity of biological therapies. This is a measure of the smallest change that can be made in the measured material that produces a response in the measuring system. Measurement variation is a factor of the equipment and its limitations, the skill of the operator, the sampling methods and the detection system. An example of this is the difference between CD34^+^ staining and flow cytometry‐gating methods such as ISHAGE, Milan and Norway techniques that can lead to different values being reported 29.
- Error and Defects – This is a common challenge within manufacturing and in this instance contributes to a number of the previous sources of variation. These are usually as a result of human *error* that leads to *defects* in the product. Examples of this could be inadvertent mistakes, surprise or a misunderstanding leading to a product that does not meet the specification 30.

## Conclusion

On the basis of the public body of knowledge, the extent of variation in therapeutic dose for HSCT is between one and four orders of magnitude of the median. It appears that this degree of variation is currently tolerated under the practice of medicine, but would be unmanageable from a biomanufacturing perspective and could affect the comparable patient outcome for HSCT. Both medical and bioengineering fields will need to share their experience in identifying the sources of variation and the strategies required to control and bring this variation within a set tolerance/therapeutic dose. Variation will never be eliminated completely, but it can be controlled to a state where the differences between products do not affect patient outcome.

Further work will include continued analysis of the data set derived from this meta‐analysis into autologous, allogeneic and paediatric subsets, and subsequently focus on high‐quality data sets such as those derived from large‐scale clinical centres and petitioned national health resources. These will begin to minimize the effect of some sources of variation (such as operator/process variety within a single centre), increase the rigour and quality of reporting and allow further stratification in terms of donor, patient and methodology. We anticipate that when this is achieved, it may be possible that the variation may be reduced from four orders of magnitude as the effects of a number of sources of variation are minimized.

## Source of Funding

Funding for this work was provided by a UK EPSRC grant (EP/FS00491/1) as part of the Doctoral Training Centre for Regenerative Medicine.

## Competing interests

The authors have no competing interests.
